# Benchmarking data of the Fair Trade USA Capture Fisheries Standard and the Marine Stewardship Council Fisheries Standard against the Food and Agricultural Organization's Voluntary Guidelines for Securing Sustainable Small-Scale Fisheries

**DOI:** 10.1016/j.dib.2019.103850

**Published:** 2019-03-23

**Authors:** Meghan E. Borland, Megan Bailey

**Affiliations:** Marine Affairs Program, Dalhousie University, NS, Canada

**Keywords:** Small-scale fisheries, Certification program, Ecolabels, Fair Trade USA, Marine Stewardship Council, Food and Agricultural Organization of the United Nations, Sustainability, Fisheries management

## Abstract

This paper presents data associated with the benchmarking of the Fair Trade USA (FT USA) Capture Fisheries Standard and the Marine Stewardship Council (MSC) Fisheries Standard against the Food and Agriculture Organization's Voluntary Guidelines for Securing Sustainable Small-Scale Fisheries in the Context of Food Security and Poverty Eradiation (FAO Voluntary Guidelines). Benchmarking was used to determine the extent to which these standards, which promote sustainability in different ways, align with the FAO Voluntary Guidelines. The data represent a comprehensive analysis of these standards and are useful for beginning to understand the appropriateness of these standards for small-scale fisheries in developing regions of the world. For further interpretation and discussion please see “A tale of two standards: A case study of the Fair Trade USA certified Maluku handline yellowfin tuna (*Thunnus albacares*) fishery” [1].

**Table:** Specifications Table

Subject area	*Small-Scale Fisheries*
More specific subject area	*Seafood Certification Programs*
Type of data	*Table*
How data was acquired	*The Compliance Criteria for the FTUSA Capture Fisheries Standard Ref #*[2], the *MSC Fisheries Standard Ref #*[3]*, and the FAO Voluntary Guidelines for Securing Sustainable Small-Scale Fisheries Ref #*[4]*were acquired online.*
Data format	*Qualitative, Benchmarking analysis*
Experimental factors	*In this paper, the FTUSA and MSC certification programs were benchmarked against the FAO Voluntary Guidelines*.
Experimental features	T*he data include both sections from the standards, as well as a stoplight ranking comparing the standards data with the FAO Voluntary Guidelines. The analysis provides an understanding of the extent to which FTUSA and MSC align with the FAO Voluntary Guidelines.*
Data source location	Input data were sourced from Ref # (2] [3], [4].This DIB presents the analysis of the data, which can be found with this article. Commentary on the data can be found in Borland, M, and Bailey, M. A tale of two standards: A case study of the Fair Trade USA certified Maluku handline yellowfin tuna (*Thunnusalbacares*) fishery [1].
Data accessibility	*Data are with this article*
Related research article	Borland M.E, Bailey M. A tale of two standards: The case of the Maluku handline yellowfin tuna fishery. Mar. Pol*.* 100: 353, 2019, 353–360.

**Table:** 

**Value of the data** • The data presented can be used by regulators to understand the extent to which the MSC and FTUSA Fisheries Standard currently align with the FAO Voluntary Guidelines, and could be used by standard holders to enhance alignment in the future • The data can provide insight for practitioners on the appropriateness of the FT USA Capture Fisheries Standard and the MSC Fisheries Standard for small-scale fisheries in developing regions of the world • The data can assist in understanding the role of the newly developed FT USA Capture Fisheries Program in relation to MSC

## 1 Data

The data presented in Table 1 shows the benchmarking of the Compliance Criteria for the FTUSA Capture Fisheries Standard and the MSC Fisheries Standard against the FAO Voluntary Guidelines. The benchmarking data consists of components of the FTUSA and MSC programs, organized by FAO Voluntary Guidelines (or sub-guideline). The benchmarking data presented in Table 1, consists of requirements of the FTUSA and MSC standards, organized by FAO guideline (or sub-guideline).

## 2 Experimental design, materials and methods

The MSC Fisheries Standard and Guidance v2.0 (MSC 2014) [3] and the FT USA Compliance Criteria for the Capture Fisheries Standard (FTUSA_CFS_CC_1.0v1_EN_121914) [2] were benchmarked against *Part 2- Responsible fisheries and sustainable development*, and *Part 3- Ensuring an enabling environment and* *supporting* *implementation*, of the FAO Voluntary Guidelines for Securing Sustainable Small-Scale Fisheries [4], both of which are divided into components.

The components are further broken into guidelines. For guidelines that were comprised of several aspects, the guidelines were further broken down into sub-guidelines. For each guideline and sub-guideline, both the FTUSA and MSC standards were assessed to determine if there were requirements or components of the certification program equivalent to the FAO Voluntary Guidelines. A stoplight methodology was used to indicate if the respective standard did or did no fulfill a guideline of the FAO Voluntary Guidelines. Green was used to indicate explicit alignment, yellow for partial fulfillment of the guideline and red if the standard did not meet the respective guideline at all. Thus the production of the benchmarking data involved the authors judging the requirements of the standards against the FAO Voluntary Guidelines. Note that criteria that were considered outside the scope of a marine certification program were ignored. For example, the FAO includes land aspects in the first guideline (5.2), but they were ignored in the analysis.
