# Supplementary material for: Benchmarking data of the Fair Trade USA Capture Fisheries Standard and the Marine Stewardship Council Fisheries Standard against the Food and Agricultural Organization's Voluntary Guidelines for Securing Sustainable Small-Scale Fisheries
Source: Data Brief. 2019 Mar 23;24:103850. doi: 10.1016/j.dib.2019.103850 (PMC6526237; doi:10.1016/j.dib.2019.103850)
Supplement: Multimedia component 2 [file mmc2.docx]

Table 1. Benchmarking of the FT USA Capture Fisheries Compliance Criteria for the Capture Fisheries Standard and the MSC Fisheries Standard against the FAO Voluntary Guidelines for Securing Sustainable Small-Scale Fisheries. Guidelines in which are outside of the scope of fisheries certification programs are coloured black. Red indicates that the certification program does not fulfill that guideline. Yellow indicates implicit alignment or partial fulfillment of the particular guideline. Green indicates explicit alignment and complete fulfillment of the guideline.

| **FAO Guideline** |  | | **Fair Trade USA (FT USA)** | **Marine Stewardship Council (MSC)** | | |
| --- | --- | --- | --- | --- | --- | --- |
| **5. Governance of tenure in small-scale fisheries and resource management** | | | | | | |
| **5a. Responsible governance of tenure** | | | | | | |
| 5.2 | All parties should recognize that responsible governance of tenure of land, fisheries and forests applicable in small-scale fisheries is central for the realization of human rights, food security, poverty eradication, sustainable livelihoods, social stability, housing security, economic growth, rural and social development. | |  | | |  |
| 5.3 | States, in accordance with their legislation, should ensure that small-scale fishers, fish workers and their communities have secure, equitable, and socially and culturally appropriate tenure rights to fishery resources (marine and inland) and small-scale fishing areas and adjacent land, with special attention paid to women with respect to tenure rights. | | Ensuring tenure rights is considered to be outside the scope of fishery certification programs. | | | |
| 5.4 | States, in accordance with their legislation, and all other parties should recognize, respect and protect all forms of legitimate tenure rights, taking into account where appropriate, customary rights to aquatic resources and land and small-scale fishing areas enjoyed by small-scale fishing communities. | | * The FT CFS does not explicitly state that legitimate tenure rights are respected and protected. However, the empowerment element of the standard contributes to fostering a sense of ownership and protection of fishing resources and areas by fishers as well as negotiation skills. | **Principle Indicator 3.1.1:** The management system exists within an appropriate and effective legal and/or customary framework which ensures that it:  - Is capable of delivering sustainability in the Unit of Assessment(s).  - Observes the legal rights created explicitly or established by custom of people dependent on fishing for food or livelihood; and incorporates an appropriate dispute resolution framework.  Scoring Issue (c): Respect for Rights | | |
|  | When necessary, in order to protect various forms of legitimate tenure rights, legislation of this effect should be provided. States should take appropriate measures to identify, record and respect legitimate tenure right holders and their rights. | | Providing legislation is considered to be outside the scope of fishery certification programs. | | | |
|  | Local norms and practices, as well as customary or otherwise preferential access to fishery resources and land by small-scale fishing communities including Indigenous peoples and ethnic minorities, should be recognized, respected and protected in ways that are consistent with international human rights law. The UN DRIP and the Declaration on the Rights of People Belonging to Religious and Linguistic Minorities should be taken into account, as appropriate. | |  | **Principle Indicator 3.1.1:** The management system exists within an appropriate and effective legal and/or customary framework which ensures that it:  - Is capable of delivering sustainability in the Unit of Assessment(s).  - Observes the legal rights created explicitly or established by custom of people dependent on fishing for food or livelihood; and incorporates an appropriate dispute resolution framework.  Scoring Issue (c): Respect for Rights  * International human rights law is not explicitly recognized in the standard nor are Indigenous peoples | | |
|  | Where constitutional or legal reforms strengthen the rights of women and place them in conflict with custom, all parties should cooperate to accommodate such changes in customary tenure systems. | | Constitutional and/or legal reforms are considered to be outside the scope of fishery certification programs. | | | |
| 5.5 | States should recognize the role of small-scale fishing communities and Indigenous peoples to restore, conserve, protect and manage local aquatic and coastal ecosystems. | | **STR-FA 1.1:** The registered fishers are members of a Fisher Association in order to ensure democratic fisher input into decision-making about changes in the management of the fishery.  **ECD-FTP 3.1:** At least 30% of the Premium is used on environmental projects that contribute to the sustainability of the fishery and/or marine ecosystem.  **RM-DC 2.3:** Data collectors, including fishers, are regularly trained in data collection, data safety (including backups), and data management.  **RM-GOV 2:** The Fisher Association is actively involved in the management of the fishery.  * It is important to note that small-scale fishing communities and Indigenous peoples cannot be equated to registered fishers in all cases. | **Principle Indicator 3.1.2**: The management system has effective consultation processes that are open to interested and affected parties.  The roles and responsibilities of organizations and individuals who are involved in the management process are clear and understood by all relevant parties.  Scoring Issue (c): Participation  **Principle Indicator 3.1.1:** The management system exists within an appropriate and effective legal and/or customary framework which ensures that it:  - Is capable of delivering sustainability in the Unit of Assessment(s)  - Observes the legal rights created explicitly or established by custom people dependent on fishing for food or livelihood, and incorporates an appropriate dispute resolution framework.  Scoring Issue (a): Compatibility of laws or standards with effective management  **Principle Indicator 3.2.2:** The fishery-specific management system includes effective decision-making processes that result in measures and strategies to achieve the objectives, and has an appropriate approach to actual disputes in the fishery.  Scoring Issue (b): (b) Responsiveness of decision-making processes  * Consultation is not considered equivalent to the direct involvement of small-scale fishing communities in restoring, conserving, protecting, and managing local aquatic and coastal ecosystems  ** It is important to note that small-scale fishing communities and Indigenous peoples cannot be equated certified fishers in all cases. | | |
| 5.6 | Where States own or control water (including fishery resources) and land resources, they should determine the use and tenure rights of these resources taking into consideration inter alia, social, economic, and environmental objectives. | | Ownership or control of water and land resources is considered to be outside of the scope of fishery certification programs. | | | |
|  | States should, as applicable, recognize and safeguard publically owned resources that are collectively used and managed, in particular by small-scale fishing communities. | | With respect to fishery resources, FT USA aims to do this and their focus is on small-scale fishing communities. | With respect to fishery resources, MSC aims to do this. However, there is not a particular emphasis on small-scale fishing communities. | | |
| 5.7 | Taking due account of Art. 6.18 of the Code, States should where appropriate grant preferential access of small-scale fisheries to fish in waters under national jurisdiction, with a view of achieving equitable outcomes for different groups of people, in particular vulnerable groups. | | Preferential access to fisheries is considered to be outside of the scope of fishery certification programs. | | | |
|  | Where appropriate, specific measures, inter alia, the creation and enforcement of exclusive zones for small-scale fisheries should be given due consideration before agreements on resource access are entered into with third countries and third parties. | | Agreements on resource access are considered to be outside the scope of fishery certification programs. | | | |
| 5.8 | States should adopt measures to facilitate equitable access to fishery resources for small-scale fishing communities including as appropriate, redistributive reform, taking into account the provisions of the Voluntary Guidelines on Responsible Governance of Tenure of Land, Fisheries and Forests in the Context of National Food Security. | | **RM-GOV 2**: The Fisher Association is actively involved in the management of the fishery.  * Fishery certification programs, including FT USA could assist in facilitating access to fishery resources by enhancing their participation in the governance of the fishery. | **Principle Indicator 3.1.2:** The management system has effective consultation processes that are open to interested and affected parties. The role and responsibilities of organizations and individuals who are involved in the management process are clear and understood by all relevant parties.  Scoring Issue (b): Consultation processes  **Principle Indicator 3.2.2:** The fishery specific management system includes effective decision-making processes that result in measures and strategies to achieve the objectives and has an appropriate approach to actual disputes in the fishery.  Scoring Issue (b): Responsiveness of decision-making processes  * Fishery certification programs, including MSC could assist in facilitating access to fishery resources by enhancing their participation in the governance of the fishery. | | |
| 5.9 | States should ensure that small-scale fishing communities are not arbitrarily evicted and that their legitimate tenure rights are not extinguished or infringed. | | Considered to be outside the scope of fishery certification programs. | | | |
|  | States should recognize that competition with other users is increasing within small-scale fisheries areas. | |  |  | | |
|  | Small-scale fishing communities, in particular vulnerable and marginalized groups, are often weaker in conflicts with other sectors and may require special support if their livelihoods are threatened by the development and activities of other sectors. | | Sectors outside of fisheries are considered outside of the scope of fishery certification programs. | | | |
| 5.10 | States and other parties should, prior to the implementation of large-scale development projects that might impact small-scale fishing communities, consider the social, economic and environmental impacts through impact studies, and hold effective and meaningful consultations with these communities, in accordance with national legislation. | | Large-scale development projects that fall outside the unit of assessment are considered to be outside of the scope of fishery certification programs. | | | |
| 5.11 | States should provide small-scale fishing communities and individuals, including vulnerable and marginalized people, access through impartial and competent judicial and administrative bodies to timely, affordable and effective means of resolving disputes over tenure in accordance with national legislation, including alternative means of resolving such disputes. | | **RM-GOV 3:** There is a procedure for resolving conflict among the certificate holder, the Fisher Association, and the legally responsible agency regarding management of the fishery and the use of its resources.  * Not specific to disputes over tenure | **Principle Indicator 3.1.1:** The management system exists within an appropriate and effective legal and/or customary framework which ensures that it:  - Is capable of delivering sustainability in the UoA(s)  - Observes the legal rights created explicitly or established by custom of people dependent on fishing for food or livelihood; and incorporates an appropriate dispute resolution framework.  Scoring Issue (b): Resolution of disputes  * Not specific to disputes over tenure | | |
|  | Provide effective remedies, which may include an entitlement appeal as appropriate. Such remedies should be promptly enforced in accordance with national legislation and may include restitution, indemnity, just compensation and reparation. | | Considered to be outside the scope of fishery certification programs and the responsibility of government authorities. | | | |
| 5.12 | States should strive to restore access to traditional fishing grounds and coastal lands to small-scale fishing communities that have been displaced by national disasters and/or armed conflict taking into consideration the sustainability of fisheries resources. | | Restoring access to traditional fishing grounds and coastal lands to small-scale fishing communities that have been displaced by national disasters and/or armed conflict is considered to be outside the scope of fishery certification programs and instead the responsibility of government authorities. | | | |
|  | States should establish mechanisms to support fishing communities affected by grave human rights violations to rebuild their lives and livelihoods. Such steps should include the elimination of any form of discrimination against women in tenure practices in case of natural disasters and/or armed conflict. | | The FT USA addresses discrimination and human rights (Section 3: Fundamental Human Rights) issues against registered fishers, potential new program participants, or workers. However, there are not requirements specific to discrimination against women. |  | | |
| **5b. Sustainable resource management** | | | | | | |
| 5.13 | | States and all those engaged in fisheries management should adopt measures for the long-term conservation and sustainable use of fisheries resources and to secure the ecological foundation for food production. | The Resource Management section of the FTUSA standard includes requirements that contribute to the long-term conservation and sustainable use of fisheries resource. | Principle Indicators under Principle 1, 2 and 3 simultaneously operate to aim to achieve the long-term conservation and sustainable use of fisheries resource. | | |
|  |  | They should promote and implement appropriate management systems, consistent with their existing obligations under national and international law and voluntary commitments, including the Code that give due recognition to the requirements and opportunities of small-scale fisheries. | - Fishery must adhere to national and international law, understanding and agreements  - FAO Code of Conduct for Responsible Fisheries  - FAO Guidelines for the Ecolabelling of Fish and Fishery Products from Marine Capture Fisheries  - Follows International Labour Organization (ILO) conventions (29, 87, 98, 100, 103, 105, 111, 138, 155, 158, 169, 182, 188) | - Fishery must adhere to national and international law, understanding and agreements  - FAO Code of Conduct for Responsible Fisheries  - Guidelines for the Ecolabelling of Fish and Fishery Products from Marine Capture Fisheries  - The Code of Good Practice for Setting - Social and Environmental Standards (ISEL)  - World Trade Organization Technical Barriers to Trade Agreement | | |
| 5.14 | | All parties should recognize that rights and responsibilities come together; tenure rights are balanced by duties, and support the long-term conservation and sustainable use of resources and the maintenance of the ecological foundation for food production | Does not explicitly state that tenure rights are balanced by duties. But collectively the criteria in the standard supports long-term conservation and sustainable use of resources and the maintenance of the ecological foundation for food production. | Does not explicitly state that tenure rights are balanced by duties. But collectively the criteria in the standard supports long-term conservation and sustainable use of resources and the maintenance of the ecological foundation for food production. | | |
|  |  | Small-scale fisheries should utilize fishing practices that minimize harm to the aquatic environment and associated species and support the sustainability of the resource. | **RM-SH 1.4:** In fisheries where ETP species are unintentionally caught, registered fishers demonstrate that they are using industry recognized best practices and available technology to avoid ETP species. This includes the use of selective fishing gear.  **RM-SH 1.6:** There is an analysis that shows the fishery does not pose a risk or serious irreversible harm to the population viability of the ETP species.  **RM-SH 2.4:** Overfishing is not occurring for any secondary or bycatch species, as determined through a peer-reviewed and tested assessment method based upon available data.  **RM-BEP 1:** The fishing gear used does not cause harm to marine habitat.  **RM-BEP 2.1:** An ecosystem monitoring strategy has been developed and implemented. Scientifically-justifiable ecosystem metrics have been identified that can be used as warning signs to changes in the ecosystem at a scale larger than the fishery. | **Principle 1: Sustainable target fish stocks**  **Principle 2: Environmental impact of fishing** | | |
| 5.15 | | States should facilitate, train and support small-scale fishing communities to participate and take responsibility for, taking into consideration their legitimate tenure rights and systems, the management of the resources on which they depend for their well-being and that are traditionally used for their livelihoods. | **STR-FA 1:** Fishers are empowered through their membership in a Fisher Association.  **RM-GOV 2:** The Fisher Association is actively involved in the management of the fishery.  **RM-DC 1.1:** A collection system is in place and accessible to all registered fishers and resource managers. This includes effective communication to illiterate members.  **RM-DC 2.3:** Data collectors, including fishers, are regularly trained in data collection, data safety (including backups) and data management. Written procedures are provided regarding the above topics.  *Does not state that their legitimate tenure rights and systems are taken into consideration. |  | | |
|  |  | Accordingly, States should involve small-scale fishing communities- with special attention to equitable participation of women, vulnerable and marginalized groups- in the design, planning, and as appropriate, implementation of protected areas, affecting their livelihood options. | Protected areas are considered to be outside the scope of fishery certification programs. | | | |
|  |  | Participatory management, such as co-management, should be promoted in accordance with national law. | **RM- GOV 2:** The Fisher Association is actively involved in the management of the fishery. | **Principle Indicator 3.1.2:** The management system has effective consultation processes that are open to interested and affected parties. The roles and responsibility of organizations and individuals who are involved in the management process are clear and understood by all relevant parties.  *Consultation is not considered equivalent to participatory management such as the involvement of the Fisher Association in FT USA | | |
| 5.16 | | States should ensure the establishment of monitoring, control and surveillance (MCS) systems or promote the application of existing ones applicable to and suitable for small-scale fisheries. They should provide support to such systems, involving small-scale fisheries actors as appropriate and promoting participatory arrangements within the context of co-management. | **STR-CH 5.1:** An internal control system has been designed and implemented. The system monitors the implementation of practices and policies mandated by the standard.  **RM- GOV 1.1:** There is no evidence that local, national, and international laws regarding fishing practices are being broken by registered fishers, including regulations concerning fishing gear, boats, fishing effort, fishing location, and illegal harvesting of species.  **RM-BEP 2.1:** An ecosystem monitoring strategy has been developed and implemented. Scientifically justifiable ecosystem metrics have been identified that can be used as warning signs to changes in the ecosystem at a scale larger than the fishery.  **RM-BEP 2.3:** Roles and responsibilities for gathering the data and recording the ecosystem metric(s) selected have been identified, with a goal of obtaining at least 6 months of data within one year. All data collected is shared with the responsible management agency.  **RM-GOV 2.1:** There has been at least one meeting in the past year between the fisheries management authorities (or their representatives) about the management regulations.  **RM-GOV 2.2:** There is a written co-management commitment signed by the Fair Trade Committee, the certificate holder, and the agency legally responsible for the resource. | **Principle Indicator 3.2.3**: Monitoring, control and surveillance mechanisms ensure the management measures in the fishery are enforced and complied with.  * Co-management is not a requirement | | |
|  |  | States should ensure effective monitoring and enforcement mechanism to deter, prevent and eliminate all forms of illegal and/or destructive fishing practices having a negative effect on marine and inland ecosystems. | **RM- BEP 1:** The fishing gear used does not cause harm to marine habitat.  **RM- BEP 2.2:** At least one ecosystem metric has been chosen to monitor and track.  **RM-GOV 1:** Illegal fishing is monitored and reported. (Includes an enforcement strategy) | **Principle Indicator 2.5.3:** There is adequate knowledge of the impacts of the UoA on the ecosystem.  Scoring Issue (e) Monitoring: Information is adequate to support the development of strategies to manage ecosystem impacts.  **Principle Indicator** **3.2.3:** Monitoring, control, and surveillance mechanisms ensure the management measures in the fishery are enforced and complied with.  Scoring Issue (b): Sanctions | | |
|  |  | States should endeavor to improve registration of fishing activity. | **STR- CH 1.6:** A list of all vessels used by registered fishers to fish Fair Trade product is kept up to date.  **STR-CH 2.1:** Vessels used by registered fishers are legally registered and licensed. | In order to comply with MSC Chain of Custody standard, this is required. | | |
|  |  | Small-scale fisheries should support the MCS systems and provide to the State fisheries authorities the information required for the management of the activity. | The FT CFS has elements of a MCS system. However unlike the MSC standard, it is not explicitly stated.  **RM-BEP 2:** Local ecosystems are monitored.  **RM-FD 2:** A Fishery Management Plan has been developed and implemented.  * The Certificate Holder is responsible for compliance with the capture fisheries standard. | **Principle Indicator 3.2.3:** Monitoring, control, and surveillance mechanisms ensure that the management measures in the fishery are enforced and complied with. | | |
| 5.17 | | States should ensure that the roles and responsibilities within the context of co-management arrangements of concerned parties and stakeholders are clarified and agreed through a participatory and legally supported process. All parties are responsible for assuming the management of roles agreed to. | **RM-GOV 2.2:** There is a written co-management commitment signed by the Fair Trade Committee, the certificate holder, and the agency legally responsible for the resource articulating:  a) A shared commitment to undertake resource management efforts necessary for Fair Trade certification.  b) A commitment to undertake collaborative management of the fishery. The fisheries management authorities will i) inform stakeholders of changes in political directives, ii) consult stakeholders prior to making decisions regarding management changes, and iii) commit to providing fair feedback to the core issues raised by stakeholders.  c) A commitment by all parties to attend at least one yearly co-management meeting aimed at collaborative discussion of management improvements, particularly those with the potential to be implemented at the scale of the fishery. | Co-management and community-based management and data collection are recognized within the MSC FS however, they are regarded as having a lower level of verifiability and higher bias than alternative data collection methods such as observer programs. | | |
|  |  | All endeavors should be made so that small-scale fisheries are represented in relevant local and national professional associations and fisheries bodies and actively take part in relevant decision-making and fisheries policy-making processes. | **STR-FA 1.1**: The registered fisher members are members of a Fishing Association in order to ensure democratic fisher input into decision-making about the changes in the management of the fishery.  **STR-FA 1.4**: All major decisions of the Fisher Association are discussed and approved by members according to a free, fair and transparent voting procedure.  **FTC** The role of the Fair Trade Committee is to ensure democratic and transparent decisions about Fair Trade issues.  **RM-GOV 2.2:** There is a written co-management commitment signed by the Fair Trade Committee, the certificate holder, and the agency legally responsible for the resource. | **Principle Indicator 3.1.2:** The management system has effective consultation processes that are open to interested and affected parties. The roles and responsibilities of organizations and individuals who are involved in the management process are clear and understood by all relevant parties  Scoring Issue (b) Consultation processes  Scoring Issue (c) Participation  **Principle Indicator 3.2.2:** The fishery specific management system includes effective decision-making processes that result in measures and strategies to achieve the objectives and has an appropriate approach to actual disputes in the fishery.  Scoring Issue (b) Responsiveness of decision-making processes.  Scoring Issue (d) Accountability and transparency of management system and decision.  *Consultation is not considered equivalent to participatory management such as the involvement of the Fisher Association in FT USA | | |
| 5.18 | | States and small-scale fisheries should encourage and support the role and involvement of both men and women in all pre-harvest, harvest and post-harvest activities, in the context of co-management and in the promotion of responsible fisheries, contributing their particular knowledge, perspectives and needs. All parties should pay specific attention to the need to ensure equitable participation of women, designing special measures to achieve this objective. | **RM-GOV 2.2:** There is a written co-management commitment signed by the Fair Trade Committee, the Certificate Holder, and the agency legally responsible for the resource.  FHR-DAP 1: There is no discrimination against registered fishers, potential new program participants, or workers.  - In the context of co-management all of the subsectors are not included |  | | |
| 5.19 | | Where transboundary and other similar issues exist, e.g. shared waters and fishery resources, States should work together to ensure that the tenure rights of small-scale fishing communities that are granted are protected. | Considered outside the scope of fishery certification programs. | | | |
| 5.20 | | States should avoid policies and financial measures that may contribute to fishing overcapacity and, hence, overexploitation of resources that have an adverse impact on small-scale fisheries. | The Fair Trade Premium is a financial measure that may contribute to overfishing however, there is a requirement specific to eliminating overfishing.  **RM - SH 2:** If overfishing is occurring, there is a strategy in place, and clear progress is being made to eliminate overfishing. | Principle 1: Sustainable target fish stocks | | |
| **6. Social development, employment and decent work** | | | | | | |
| 6.1 | | All parties should consider integrated, ecosystem and holistic approaches to small-scale fisheries management and development that take the complexity of livelihoods into account. | **Operational objectives:**  1) Empowerment  2) Economic Development  3) Social Responsibility  4) Environmental Stewardship | The MSC Standard is an ecological standard. | | |
|  |  | Due attention to social and economic development may be needed to ensure that small-scale fishing communities are empowered and can enjoy their human rights. | **Operational Objectives:**  2) Economic Development  3) Social Responsibility |  | | |
| 6.2 | | States should promote investment in human resource development such as health, education, literacy, digital inclusion and other skills of technical nature that generate added value to the fisheries resources as well as awareness raising. | Fishers are encouraged to use their Fair Trade Premium to provide greater access to, or improved quality of, health care and education (Fair Trade USA, 2014).  **RM-DC 2.3:** Data collectors, including fishers, are regularly trained in data collection, data safety (including backups) and data management. Written procedures are provided regarding the above topics. |  | | |
|  |  | States should take steps with a view to progressively ensure that members of small-scale fishing communities have affordable access to these and other essential services trough national and subnational actions, including adequate housing, basic sanitation that is safe and hygienic, safe drinking water for personal and domestic uses, and sources of energy. | Considered to be outside the scope of fishery certification programs. | | | |
|  |  | Preferential treatment of women, Indigenous peoples, and vulnerable and marginalized groups- in providing services and giving effect to non-discrimination and other human rights- should be accepted and promoted where it is required to ensure equitable benefits. | **FHR-DAP 1:** There is no discrimination against registered fishers, potential new program participants, or workers.  **FHR-DAP 1.3:** Where applicable, a program has been developed to improve the social and economic position of registered fishers who come from disadvantaged/minority groups. |  | | |
| 6.3 | | States should promote social security protection for workers in small-scale fisheries. | **WWS- CE 5.1:** The employer complies with local law regarding the payment and provision of social services including social security, pension, and health and disability insurance. |  | | |
|  |  | They should take into account the characteristics of small-scale fisheries and apply security schemes to the entire value chain. | **WWS-CE 5:** The employer complies with local law regarding the provision of social security, pension, and health and disability insurance. In cases where permanent workers are not entitled to health insurance benefits, the employer provides equivalent benefits in the form of private health insurance or comparable health services.  * The degree of inclusivity of activities along the value chain is case specific. |  | | |
| 6.4 | | States should support the development of and access to other services that are appropriate for small-scale fishing communities with regard to, for example, savings, credit and insurance schemes, with special emphasis on ensuring the access of women to such services. | **WWS- CE 5.1**: The employer complies with local law regarding the payment and provision of social services including social security, pension, and health and disability insurance.  **WWS-CE 5.2:** In cases where permanent workers are not entitled to health insurance benefits, the employer provides the equivalent benefits in the form of private health insurance or comparable health services.  **ECD-DM 2.2:** The Fair Trade Premium Plan contains a reasonable budget based upon expected Fair Trade Premium income. |  | | |
| 6.5 | | States should recognize as economic and professional operations the full range of activities along the small-scale fisheries value chain- both pre and post-harvest: whether undertaken on water or on land; undertaken by men or by women. | The degree of inclusivity of activities along the value chain is case specific. It depends on which workers are employed by the Certificate Holder or individual fishers. | The MSC Fisheries Standard focuses on the harvest sector. The MSC Chain of Custody standard does include aspects of the post-harvest sector. | | |
|  |  | All activities should be considered: part-time, occasional and/or for subsistence. Professional and organizational development opportunities should be promoted in particular for more vulnerable groups of post-harvest fish works and women in small-scale fisheries. | **RM-DC 2.3:** Data collectors, including fishers, are regularly trained in data collection, data safety (including backups) and data management. Written procedures are provided regarding the above topics.  Promoted in the harvest subsector but varies within the post-harvest subsector based on the fishery of interest. |  | | |
| 6.6 | | States should promote decent work for all small-scale fisheries workers, including both the informal and formal sectors. | **WWS-CE:** Workers have clear employment conditions, and all conditions are respected by the employer.  **WWS-OH:** Workplace risks are minimized and employers take all appropriate measures to ensure they and their employees are safe from harm. |  | | |
|  |  | States should create the appropriate conditions to ensure that fisheries activities in both the formal and informal sectors are taken into account in order to ensure the sustainability of small-scale fisheries in accordance with national law. | - All Fair Trade Certified producers, fishermen, and Certificate Holders are expected to comply with all relevant local and national laws and regulations  - Activities in the informal sector are not included | **Principle 3: Effective Management**  - Activities in the informal sector are not included | | |
| 6.7 | | States should take steps with a view to the progressive realization of the right of small-scale fishers and fish workers to an adequate standard of living and work in accordance with national and international human rights standards. States should create an enabling environment for sustainable development in small-scale fishing communities. | **WWS: Wages, Working Conditions & Access to Services** |  | | |
|  |  | States should pursue inclusive, non-discriminatory, and sound economic policies for the use of marine, freshwater and land areas in order to permit small-scale fishing communities and other producers, particularly women, to earn a fair return from their labour, capital and management, and encourage conservation and sustainable management of natural resources. | **FHR-DAP 1.1:** There is no discrimination against registered fishers or potential new program participants, particularly on the basis of race, color, sex, gender, sexual orientation, disability, marital status, family obligations, age, religion, political opinion, language, property, nationality, ethnicity or social origin, or any other condition that could give rise to discrimination in relation to: participation, rules for program participation, voting rights, the right to be elected, access to markets, access to training, technical support, or any other benefits the program offers.  FHR-DAP 1.2: There is no discrimination against workers, particularly on the basis of race, color, sex, gender, sexual orientation, disability, marital status, family obligations, age, religion, political opinion, language, property, nationality, ethnicity or social origin, membership of unions or other workers’ representative bodies, or any other condition that could give rise to discrimination in: recruitment, promotion, access to training, remuneration, allocation of work, termination of employment, retirement, or other activities.  FHR - DAP 1.3: Where applicable, a program has been developed to improve the social and economic position of registered fishers who come from disadvantaged/minority groups. |  | | |
| 6.8 | | States and other stakeholders should support already existing, or the development of complementary and alternative income generating opportunities- in addition to earnings from fisheries-related activities- for small scale fishing communities, as required and in support of sustainable resource utilization and livelihood diversification. | Activities outside of fishing are considered to be outside the scope of fishery certification programs | | | |
|  |  | The role of small-scale fisheries in local economies and the links of the subsector to the wider economy need to be recognized and benefited from. | Program developed to be delivered to small-scale fisheries and therefore their link to the wider economy is recognized. | Small-scale fisheries can go through the MSC progress and thus their link to the wider economy is recognized and may be benefited from. | | |
|  |  | Small-scale fishing communities should equitably benefit from developments such as community-based tourism and small-scale responsible aquaculture. | Activities outside of fishing are considered to be outside the scope of fishery certification programs. | | | |
| 6.9 | | All parties should create conditions for men and women of small-scale fishing communities to fish and to carry out fisheries-related activities in an environment free of crime, violence, organized crime activities, piracy, theft, sexual abuse, corruption, and abuse of authority. | **FHR- DAP 1.1:**  There is no discrimination against registered fishers or potential new program participants, particularly on the basis of race, color, sex, gender, sexual orientation, disability, marital status, family obligations, age, religion, political opinion, language, property, nationality, ethnicity or social origin, or any other condition that could give rise to discrimination in relation to: participation, rules for program participation, voting rights, the right to be elected, access to markets, access to training, technical support, or any other benefits the program offers.  **FHR-DAP 1.2:** There is no discrimination against workers, particularly on the basis of race, color, sex, gender, sexual orientation, disability, marital status, family obligations, age, religion, political opinion, language, property, nationality, ethnicity or social origin, membership of unions or other workers’ representative bodies, or any other condition that could give rise to discrimination in: recruitment, promotion, access to training, remuneration, allocation of work, termination of employment, retirement, or other activities.  **FHR-FL 1:** Human trafficking and forced, bonded, and compulsory labor does not occur.  **FHR-PC 1:** Children below the age of 15 (or below the working age defined by national law, if higher) are not employed anywhere in the operation. The minimum age for employment on fishing vessels is 16 or as defined in law, if higher.  **FHR-FR 1:** Freedom of association is respected and workers can freely organize. |  | | |
|  |  | All parties should take steps to institute measures that aim to eliminate violence and to protect women exposed to such violence in small-scale fisheries communities. | Section 3 of the FTUSA Fisheries standard focuses on Fundamental Human Rights (FHR). Sub-sections:  - Discrimination and Abuse Prevention (DAP)  - Freedom from Forced Labor and Human - Trafficking (FL)  - Protection of Child and Young Persons (PC)  - Freedom of Association (FR) |  | | |
|  |  | States should ensure access to justice for victims of inter alia violence and abuse, including within the household or community. | Considered to be outside the scope of fishery certification programs. | | | |
|  |  | States and small-scale fisheries actors, including traditional and customary authorities, should understand, recognize and respect the role of migrant fishers and fish workers in small-scale fisheries, given that migration is a common livelihood strategy in small-scale fisheries. States and small-scale fisheries actors should create appropriate frameworks to allow for fair and adequate integration of migrants who engage in sustainable use of fisheries resources and who do not undermine local community-based fisheries governance and development in small-scale fisheries in accordance with national law. | **ECD- DM 1.1:** The certificate holder has conducted or financed a written needs assessment using surveys/input from fishers as the primary data source, identifying the social, economic and environmental development needs of the registered fishers, workers, community, and natural resources  Interpretation Guidance: Surveys or other primary data sources must be representative of the entire population of registered fishers, community members, and workforce, including migrant and/or seasonal  workers.  **ECD- DM 1.3:** If there are workers under the scope of the certificate, their needs are taken into account in the needs assessment, focusing especially on those groups of workers that form a majority of the workforce.  Interpretation Guidance: Clarification: If migrant workers form the majority of the workforce, the needs assessment  must focus on their needs.  **ECD- DM 2.9:** The Fair Trade Premium Plan includes at least one project or activity intended to benefit workers.  Interpretation Guidance: The activity should benefit all workers and focuses on those groups of workers that form majority of the workforce (i.e., if migrant workers form the majority of the work force, the Fair Trade Premium Plan should focus on their needs). |  | | |
|  |  | States should recognize the importance of coordinating among their respective national governments in regard to migration of fishers and fish workers in small-scale fisheries across national borders. Policies and management measures should be determined in consultation with small-scale fisheries organizations and institutions. | Considered to be outside the scope of fishery certification programs as this is the role of the national government. | | | |
| 6.11 | | States should recognize and address the underlying causes and consequences of transboundary movement of fishers and contribute to the understanding of transboundary issues affecting the sustainability of small-scale fisheries. |  | **SA4.1.1** Teams shall determine and state which jurisdictional category or combination of jurisdictional categories, apply to the management system of the unit of assessment, when assessing performance of units of assessment under principle 3.  - No specific attention to the underlying causes and consequences but consideration is given. | | |
| 6.12 | | States should address occupational health issues and unfair working conditions of all small-scale fishers and fish workers by ensuring that the necessary legislation is in place and is implemented in accordance with national legislation and international human rights standards and international instruments to which the State is a contracting party, such as the International Covenant on Economic, Social and Cultural Rights (ICESCR) and relevant conventions of the International Labour Organization (ILO). | Considered to be outside the scope of fishery certification programs. | | | |
|  |  | All parties should strive to ensure that occupational health and safety is an integral part of fisheries management and development initiatives. | **WWS- OH:** Wages, Working Conditions and Access to Services- Occupational Health and Safety |  | | |
| 6.13 | | States should eradicate forced labour, prevent debt-bondage of women, men and children, and adopt effective measures to protect fishers and fish workers, including migrants, with a view to eliminate forced labour in fisheries including small-scale fisheries. | **FHR-FL 1.1:** Human trafficking and forced, bonded, and compulsory labor does not occur.  **FHR- FL 1.3:** The employer does not retain any part of the workers’ salary, benefits, property or documents in order to force them to remain.  **FHR-1.5:** Bonded labor caused by debts or loans does not occur. | Companies, which have been successfully prosecuted for, faced labour violations in the last two years not eligible for MSC certification (MSC, 2014).  “As of August 2018, as part of the MSC assessment, fisheries and offshore supply chains will be required to provide a self-disclosure document that reports on measures, policies and practices in place to ensure absence of forced and child labour” (MSC, 2018) | | |
| 6.14 | | States should provide and enable access to schools and education facilities that meet the needs of small-scale fishing communities and that facilitate gainful and decent employment of youth, respecting their career choices and providing equal opportunities for all boys and girls and young men and women. | Potential investment for the Fair Trade Premium |  | | |
| 6.15 | | Small-scale fisheries actors should recognize the importance of children’s well-being and education for the future of the children themselves and of society at large. Children should go to school, be protected from all abuse and have all their rights respected in accordance with the Convention on the Rights of the Child. | **FHR-PC 2.1:** If children of the registered fishers below the age of 15 (or below the working age defined by national law, if higher) help their relatives with work after school and/or during holidays, the work does not jeopardize the child’s social, moral, or physical development, constitute a hazard to the child’s health, jeopardize schooling, and is within reasonable time limits after school or during holidays. A relative or legal guardian supervises and guides the child. |  | | |
| 6.16 | | All parties should recognize the complexity that surrounds safety at sea issues (in inland and marine fisheries) and the multiple causes behind deficient safety. This applies to all fishing activities. | **WWS-OH 1.3:** Registered fishers and applicable workers use Personal Protective Equipment (PPE) as appropriate to their task. The PPE is functional and properly maintained, and registered fishers and applicable workers have been trained on proper use. PPE is not taken home.  Interpretation guidance: PPE includes life jackets.  **WWS- OH 3:** Individuals have the training and information they need to keep themselves safe.  **WWS- OH 4:** Policies and procedures are in place to promote health and safety in the workplace.  * Does not explicitly state or provide background on the multiple causes behind deficient safety |  | | |
|  |  | States should ensure the development, enactment and implementation of appropriate national laws and regulations that are consistent with international guidelines of FAO, the ILO and the International Maritimes Organization (IMO) for work in fishing and sea safety in small-scale fisheries. | Follows ILO  FAO Code of Conduct for Responsible Fisheries | FAO Code of Conduct for Responsible Fisheries | | |
| 6.17 | | States should recognize that improved sea safety, which includes occupational health and safety, in small-scale fisheries (inland and marine) will best be achieved through the development and implementation of coherent and integrated national strategies, with the active participation of fishers themselves and with elements of regional coordination, as appropriate. | Considered to be outside the scope of fishery certification programs as this would be the responsibility of the national government. | | | |
|  |  | Safety at sea of small-scale fishers should also be integrated into the general management of fisheries. | Occupational Health and Safety (OH) section of the standard. | There is no explicit statement of the integration of safety at sea within management plan however, measures for minimizing unwanted catch should not be implemented that adversely affect crew safety. | | |
|  |  | States should provide support to, among other things, maintenance of accident reporting, provision of sea safety awareness programmes. | **WWS-OH 4.5:** Incident reports are summarized annually and submitted to relevant authorities to provide notification/statistics on fatalities, injuries and diseases. |  | | |
|  |  | Introduction of appropriate legislation for sea safety in small-scale fisheries | The introduction of legislation is outside of the scope of seafood certification programs. | | | |
|  |  | The role of existing institutions and community-based structures for increasing compliance, data collection, training and awareness and search and rescue operations should be recognized in this process | **Occupational Healthy and Safety (OH)**  **WWS- OH 4.6:** An electronic communications network for finding lost vessels and coordinating ship to shore communications is in place on all vessels. |  | | |
|  |  | States should promote access to information and to emergency location systems for rescue at sea for small-scale vessels. | **WWS-OH 4.6:** An electronic communications network for finding lost vessels for finding lost vessels and coordinating ship to shore communications is in place. |  | | |
| 6.18 | | Taking into account the Voluntary Guidelines of the Responsible Governance of Tenure, Fisheries and Forests in the Context of National Food Security including section 25, all parties should protect the human rights and dignity of small-scale fisheries stakeholders in situations of armed conflict in accordance with international humanitarian law to allow them to pursue their traditional livelihoods, to have access to customary fishing grounds and to preserve their culture and way of life. Their effective participation in decision-making on matters that impact them should be facilitated. | Situations of armed conflict are considered outside the scope of seafood certification programs. | | | |
| **Value chains, post-harvest and trade** | | | | | | |
| 7.1 | | All parties should recognize the central role that the small-scale fisheries post-harvest subsector and its actors play in the value chain. | Not explicitly recognized, however the Product Traceability sub-section under the Traceability and Transparency section of the standard recognizes the post-harvest sector.  - The degree of inclusivity of activities under the Certificate is case specific to the fishery under assessment. The scope of the Certificate can cover a processing facility buying from one or more groups of vessels. | Not explicitly recognized however, the MSC Chain of Custody Standard recognizes the post-harvest sector with respect to traceability. | | |
|  |  | All parties should ensure that post-harvest actors are part of relevant decision-making processes, recognizing that there are sometimes unequal power relationship between value chain actors and that vulnerable and marginalized groups may require special support. |  |  | | |
| 7.2 | | All parties should recognize the central role women often play in the post-harvest subsector and support improvements to facilitate women’s participation in such work. | The scope of the Certificate can vary between fisheries under assessment. If the post-harvest subsector is employed by the certificate holder:  FHR - DAP 1: There is no discrimination against registered fishers, potential new program participants, or workers. |  | | |
|  |  | States should ensure that amenities and services appropriate for women are available as required in order to enable women to retain and enhance their livelihoods in the post-harvest subsector. | The scope of the Certificate can vary between fisheries under assessment. If the post-harvest subsector is employed by the Certificate Holder the following guidelines would apply:  **WWS-OH 2.4:** Where a significant number of workers are employed, sanitary facilities are provided separately for men and women.  **FHR-DAP 1.3:** Where applicable, a program has been developed to improve the social and economic position of registered fishers who come from disadvantaged/minority groups.  Interpretation Guidance:  - Encouraging Fair Trade Committee to adopt a constitutional clause requiring that one project per year is designed by traditionally disadvantaged (e.g., women, Indigenous, minority) populations for their benefit. This would involve consulting them directly about their own needs.  - Focusing on the needs of disadvantaged groups and communities when developing the Fair Trade Premium Plan, for instance mapping access to services or assessing quality of available services.  * Amenities are services are not ensured but encouraged |  | | |
| 7.3 | | States should foster, provide and enable investments in appropriate infrastructures, organization structures and capacity development to support the small-scale fisheries post-harvest subsector in producing good quality and safe fish and fishery products, for both export and domestic markets, in a responsible and sustainable manner. | **WWS-OH 1.8:** A maintenance and repair system is put in place to ensure safe, clean and hygienic environment at all time.  Interpretation Criteria: This criterion applies to all workplace sites, e.g., vessels, docks, landing sites, and processing locations.  * The post-harvest sector is not the focus and this seafood certification program and in some cases, neither is the domestic market. |  | | |
| 7.4 | | States and development partners should recognize the traditional forms of associations or fishers and fish workers and promote their adequate organizational and capacity development in all stages of the value chain in order to enhance their income and livelihood security in accordance with national legislation. | FT USA promotes the adequate organizational capacity of fishers through the mandatory formation of Fisher Associations and General Assembly meetings. This is not necessarily a traditional form of association and does not include all fish workers. |  | | |
|  |  | Accordingly, there should be support for the setting up and development of cooperatives, professional organizations of the small-scale fisheries sector and other organization structures, as well as marketing mechanisms, e.g. auctions, as appropriate. | FA: Fisher Association  FTC: Fair Trade Committee  FT USA acts as a marketing mechanism | MSC acts as a marketing mechanism but it does not provide support or the setting up and development of organizational structures. | | |
| 7.5 | | All parties should avoid post-harvest losses and waste and seek ways to create value addition, building also on existing traditional and local cost-efficient technologies, local innovations and culturally appropriate technology transfers. |  |  | | |
|  |  | Environmentally sustainable practices within an ecosystem approach should be promoted, deterring, for example, waster of inputs (water, fuelwood, etc.) in small-scale fish handling and processing. | **RM-WM 1:** Waste disposal does not threaten human health or the environment. |  | | |
| 7.6 | | States should facilitate access to local, national, regional and international markets. | Depending on the location of the certified fishery, access to local, national, regional and international markets may be improved upon certification. In countries such as Indonesia, certification would likely facilitate access to international markets, but not local markets as demand for certified products does not currently exist.  **STR- CH 4.6:**  The certificate holder and Fisher Association take measures to improve the registered fishers’ understanding of financial management and to increase their knowledge on pricing and international market mechanisms. | Depending on the location of the certified fishery, access to local, national, regional and international markets may be improved upon certification. In countries such as Indonesia, certification would likely facilitate access to international markets, but not local markets as demand for certified products does not currently exist. | | |
|  |  | Promote equitable and non-discriminatory trade for small-scale fisheries products. | Not all small-scale fisheries have the resources or capital to undertake fishery certification. | Not all small-scale fisheries have the resources or capital to undertake fishery certification. | | |
|  | | States should work together to introduce trade regulations and procedures that in particular support regional trade in products from small-scale fisheries and taking into account agreements under the World Trade Organization (WTO), bearing in mind the rights and obligations of WTO members where appropriate. | Considered outside the scope of fishery certification programs. | | | |
| 7.7 | | States should give due consideration to the impact of international trade in fish and fishery products and of vertical integration on local small-scale fishers, fish workers and their communities. |  |  | | |
|  |  | States should ensure that promotion of international fish trade and export production do not adversely affect the nutritional needs of the people for whom fish is critical to a nutritious diet, their health and well-being and for whom other comparable sources of food are not readily available or affordable. |  | **Principle Indicator 3.1.1:** The management system exists within an appropriate and effective legal and/or customary framework which ensures that it: - Is capable of delivering sustainability in the UoA(s) - Observes the legal rights created explicitly or established by custom of people dependent on fishing for food or livelihood; and  - Incorporates an appropriate dispute resolution framework.  - Scoring Issue (c): (c) Respect for rights  * Follows legal rights established by custom on people dependant on fishing for food or livelihood but MSC does not actively work to ensure that that international fish trade and export does not adversely affect them. | | |
| 7.8 | | States, small-scale fisheries actors and other value chain actors should recognize that benefits from international trade should be fairly distributed. | **ECD - DM 1:** There is a written assessment that outlines the needs of the fishers, workers, community, and the environment.  **ECD - DM 2:** There is a Fair Trade Premium Plan which details how the Fair Trade Premium will be used to address the needs of the registered fishers, workers, community, and/or environment.  * With respect to the FT USA Premium the benefits appear to fairly distributed |  | | |
|  |  | States should ensure that effective fisheries management systems are in place to prevent overexploitation driven by market demand that can threaten the sustainability of fisheries resources, food security and nutrition. | **RM-RD 2:** A Fishery Management Plan has been developed and implemented.  **RM- SH 2:** If overfishing is occurring, there is a strategy in place, and clear progress is being made to eliminate overfishing. | **Principle 3: Effective Management** | | |
|  |  | Such fisheries management systems should include responsible post-harvest practices, policies and actions to enable export income to benefit small-scale fishers and others in an equitable manner throughout the value chain. | **RM-WM 1.1:** The processing facilities’ procedures for handling waste are in line with applicable laws and regulations.  **RM-WM 1.4:** Wastewater from processing facilities is handled in a manner that does not have a negative impact on water quality, the environment, and/or human health.  The Fair Trade Premium enables fishing communities to benefit from export income. However, depending on the scope of the certificate, the post-harvest sector is not always included. |  | | |
| 7.9 | | States should adopt policies and procedures, including environmental, social, and other relevant assessments, to ensure that adverse impacts of international trade on the environment, small-scale fisheries culture, livelihoods, and special needs related to food security are equitably addressed. | The FT USA standard includes both environmental and social criteria. It also provides a Fair Trade Premium that can reduce the adverse impacts of international trade on small-scale fishing communities. | MSC aims to reduce the negative impacts of fisheries on the environment. The MSC Fisheries standard does not consider the social, cultural, and livelihood impacts of international trade on small-scale fishing communities. | | |
|  |  | Consultation with concerned stakeholders should be part of these policies and procedures. | **STR-FTC 2.2:** The minutes of Fair Trade Committee meetings clearly record all decisions made, as well as the consultation with registered fishers that took place prior to decision-making.  **STR-CH 4.2:** There are regular meetings between the certificate holder and the Fisher Association(s) concerning the Fair Trade program and management of the fishery. Issues and concerns of the registered fishers and applicable workers are solicited and discussed. These meetings are documented.  **STR-FA 1.1:** The registered fishers are members of a Fisher Association in order to ensure democratic fisher input into decision-making about changes in the management of the fishery.  **STR-FA 1.3:** The internal rules of the Fisher Association (i.e., statutes, constitution, or by-laws) mandate that all members have decision-making voting rights.  **STR-FA 1.4:** All major decisions of the Fisher Association are discussed and approved by members according to a free, fair, and transparent voting procedure.  **RM-GOV 2.1:** There has been at least one meeting in the past year between the fisheries management authorities and registered fishers (or their representatives) about the main management regulations.  **RM-GOV 2.2:** There is a written co-management commitment signed by the Fair Trade Committee, the certificate holder, and the agency legally responsible for the resource    - Consultation but not specifically related to the adverse effects of international trade | **Principle Indicator 3.1.2:** The management system has effective consultation processes that are open to interested and affected parties. The roles and responsibilities of organizations and individuals who are involved in the management process are clear and understood by all relevant parties.  Scoring Issue (b) Consultation processes  Scoring Issue (c) Participation  **Principle Indicator 3.2.2**  The fishery- specific management system includes effective decision- making processes that result in measures and strategies to achieve the objectives and has an appropriate approach to actual disputes in the fishery.  Scoring Issue (b) Responsiveness of decision-making process  Scoring Issue (d) Accountability and transparency of management system and decision making process  - Consultation but not specifically related to the adverse effects of international trade | | |
| 7.10 | | States should enable access to all relevant market and trade information for stakeholders in the small-scale fisheries value chain. | **STR-CH 4.6** The certificate holder and Fisher Association take measures to improve the registered fishers’ understanding of financial management and to increase their knowledge on pricing and international market mechanisms.  **ECD-FTP 4:** Communication about Fair Trade sales and use of the Fair Trade Premium is thorough and consistent.  * The degree of inclusivity of stakeholders is case specific to the fishery under assessment. |  | | |
|  |  | Small-scale fisheries stakeholders must be able to access timely and accurate market information to help them adjust to changing market conditions |  |  | | |
|  |  | Capacity development is also required so that all small-scale fisheries stakeholders, especially women and vulnerable and marginalized groups can adapt to, and benefit equitably from, opportunities of global market trends and local situations while minimizing any potential negative impacts. | **STR-CH 4.3:** If the Fisher Association wants to take on additional responsibilities for the production and commercialization of the product and wants to become certified against the Capture Fishery Standard independent of the certificate holder, the certificate holder does not prevent this development.  **STR-CH 4.6** The certificate holder and Fisher Association take measures to improve the registered fishers’ understanding of financial management and to increase their knowledge on pricing and international market mechanisms. **STR-FA 2.6:** All records, books, and documentation are accessible to members of the Fisher Association. This includes effective communication to illiterate members.  **ECD-DM 1**: There is a written needs assessment that outlines the needs of the fishers, works, community, and the environment.  **ECD-DM 2:** There is a Fair Trade Premium Plan which details how the Fair Trade Premium will be used to address the needs of registered fishers, workers, community, and/or environment  **FHR-DAP 1.3:** Where applicable, a program has been developed to improve the social and economic position of registered fishers who came from disadvantaged/minority groups.  - Not an emphasis on women, and vulnerable and marginalized groups | MSC Capacity Building Program which includes the MSC Capacity Building Toolkit  - Not an emphasis on women, and vulnerable and marginalized groups | | |
| **Gender Equity** | | | | | | |
| 8.1 | | All parties should recognize that achieving gender equality requires concerted efforts by all and that gender mainstreaming should be an integral part of all small-scale fisheries development strategies. |  |  | | |
|  |  | These strategies to achieve gender equality require different approaches in different cultural contexts and should challenge practices that are discriminatory against women. | **FHR-DAP 1:** There is no discrimination against registered fishers, potential new program participants, or workers. |  | | |
| 8.2 | | States should comply with their obligations under international human rights law and implement the relevant instruments to which they are a party including, inter alia, CEDAW, and should bear in mind the Beijing Declaration and Platform for Action. | Considered to be outside the scope of fishery certification programs. | | | |
|  |  | States should endeavor to secure women’s equal participation in decision-making processes for policies directed towards small-scale fisheries. | **FHR-DAP 1.1:** There is no discrimination against registered fishers or potential new program participants, particularly on the basis of race, color, sex, gender, sexual orientation, disability, marital status, family obligations, age, religion, political opinion, language, property, nationality, ethnicity or social origin, or any other condition that could give rise to discrimination in relation to: participation, rules for program participation, voting rights, the right to be elected, access to markets, access to training, technical support, or any other benefits the program offers.  **FHR-DAP 1.3:** Where applicable, a program has been developed to improve the social and economic position of registered fishers who came from disadvantaged/minority groups. |  | | |
|  |  | States should adopt specific measures to address discrimination against women while creating spaces for Civil Society Organizations (CSOs), in particular for women fish workers and their organizations, to participate in their implementation. | **FHR-DAP 1.1:** There is no discrimination against registered fishers or potential new program participants, particularly on the basis of race, color, sex, gender, sexual orientation, disability, marital status, family obligations, age, religion, political opinion, language, property, nationality, ethnicity or social origin, or any other condition that could give rise to discrimination in relation to: participation, rules for program participation, voting rights, the right to be elected, access to markets, access to training, technical support, or any other benefits the program offers.  **FHR-DAP 1.2:** There is no discrimination against workers, particularly on the basis of race, color, sex, gender, sexual orientation, disability, marital status, family obligations, age, religion, political opinion, language, property, nationality, ethnicity or social origin, membership of unions or other workers’ representative bodies, or any other condition that could give rise to discrimination in: recruitment, promotion, access to training, remuneration, allocation of work, termination of employment, retirement, or other activities  *No explicit mention of the creation of CSO’s, in particular for women fish workers and their organizations, to participate in their implementation (Aside from Fisher Associations) |  | | |
|  |  | Women should be encourages to participate in fisheries organizations, and relevant organizational development should be provided. | . |  | | |
| 8.3 | | State should establish policies and legislation to realize gender equality, and, as appropriate, adapt legislation, policies and measures that are not compatible with gender equality, taking into account social, economic and cultural aspects. States should be at the forefront of implementing actions for achieving gender equality by inter alia, recruiting both men and women as extension staff and ensuring that both men and women have equal access to extension and technical services, including legal support, related to fisheries. | The establishment of policies and legislation is considered outside the scope of seafood certification programs. | | | |
|  |  | All parties should collaborate to develop functional evaluation systems to assess the impact of legislation, policies and actions for improving women’s status and achieving gender equality. |  |  | | |
| 8.4 | | All parties should encourage development of better technologies of importance and appropriate to women’s work in small-scale fisheries. |  |  | | |
| **9. Disaster risks and climate change** | | | | | | |
| 9.1 | | States should recognize that combating climate change, including in the context of small-scale fisheries requires urgent and ambitious action in accordance with United Nations Framework Convention on Climate Change (UNFCCC), taking into account RIO+20 output document “The Future We Want” |  |  | | |
| 9.2 | | All parties should recognize and take into account the differential impact of natural and human-induced disasters and climate change on small-scale fisheries |  | **Principle Indicator 2.5.3:** There is adequate knowledge of the impacts of the Unit of Assessment (UoA) on the ecosystem.  Scoring Issue (b): Investigation of UoA impacts  SA3.18.1.2 Focus on the “main interactions between the UoA and these ecosystem elements” at the SG100 level. At this level:   1. UoAs should be capable of adapting management to environmental changes as well as managing the effect of the UoA on the ecosystem. 2. Monitoring the effects of environmental change on the natural productivity of the UoAs should be considered best practice and should include recognition of the increasing importance of climate change.   **GSA2.2.7:** MSC recognizes that the productivity of fisheries is affected by a range of environmental factors, as much as by the levels of fishing and the management of the fishery. The actual values of reference points may thus change over time as reflected in stock assessments, and these changes may be allowed for in scoring the status of the stock in Principle Indicator 1.1.1. In situations where there is evidence that productivity changes are related to the impacts of long-term climate change, CABs should that appropriate adjustments need to be made to reference points and indicators used to determine stock status.  **SA2.2.7.1:** If changes in fishery productivity are due to natural environmental fluctuations, teams shall accept adjustments to the reference points consistent with such natural environmental fluctuations. (Climate change is considered as a natural environmental fluctuation as it is not a human-induced impact that can be easily resolved) | | |
|  |  | States should develop policies and plans to address climate change in fisheries, in particular strategies for adaptation and mitigation and building resilience, in full and effective consultation with fishing communities including Indigenous peoples, men and women and paying particular attention to vulnerable and marginalized groups. |  |  | | |
|  |  | Special support should be given to small-scale fishing communities living on small islands where climate change may have particular implications for food security, nutrition, housing and livelihoods. |  |  | | |
| 9.3 | | All parties should recognize the need for integrated and holistic approaches, including cross-sectoral collaboration, in order to address disaster risks and climate change in small-scale fisheries. | Cross-sectoral collaboration is considered outside the scope of fishery certification programs. | | | |
|  |  | States and other relevant parties should take steps to address issues such as pollution, coastal erosion and destruction of coastal habitats due to human-induced non-fisheries-related factors. Such concerns seriously undermine the livelihoods of fishing communities as well as their ability to adapt to possible impacts of climate change. | **ECD-FTP 3.1:** At least 30% of the Premium is used on environmental projects (sustainability of the fishery and/or the marine ecosystem). |  | | |
| 9.4 | | States should consider assisting and supporting small-scale fishing communities affected by climate change or natural and human-induced disasters, including through adaptation, mitigation and aid plans, where appropriate. | The wider impact climate change and disasters is considered to be outside the scope of seafood certification programs. However, it should be noted that these impacts could be addressed in the Fair Trade program through the Fair Trade Premium. | | | |
| 9.5 | | In case of disasters caused by humans impacting small-scale fisheries, the responsible party should be held accountable. | Considered to be outside the scope of fishery certification programs. | | | |
| 9.6 | | All parties should take into account the impact that climate change and disasters may have on the post-harvest and trade subsector in the form of changes in fish species and quantities, fish quality and shelf life, and implications with regard to market outlets. States should provide support to small-scale fisheries stakeholders with regard to adjustment measures in order to reduce negative impacts. |  |  | | |
|  |  | When new technologies are introduced, they need to be flexible and adaptive to future changes in species, products and markets and climate variability. |  |  | | |
| 9.7 | | States should understand how emergency response and disaster preparedness are related in small-scale fisheries and apply the concept of the relief-development continuum. | Considered to be outside the scope of seafood certification programs. | | | |
|  |  | Longer-term development objectives need to be considered throughout the emergency sequence, including in the immediate relief phase, and rehabilitation, reconstruction and recovery should include actions to reduce vulnerability to potential future threats. | Considered to be outside the scope of seafood certification programs. | | | |
|  |  | The concept of “building back better” should be applied in disaster response and rehabilitation. | Considered to be outside the scope of seafood certification programs. | | | |
| 9.8 | | All parties should promote the role of small-scale fisheries in efforts related to climate change. |  |  | | |
|  |  | Encourage and support energy efficiency in the subsector, including the whole value chain- fishing, post-harvest, marketing and distribution. |  |  | | |
| 9.9 | | States should consider making available to small-scale fishing communities transparent access to adaptation funds, facilities and/or culturally appropriate technologies for climate change adaptation. | The Fair Trade Premium fund could be used for projects or technologies related to climate change adaptation. |  | | |
| **10. Policy Coherence, institutional organization and collaboration** | | | | | | |
| 10.1 | | States should recognize the need for and work towards policy coherence with regard to, inter alia: national legislation; international human rights law; other international instruments, including those related to Indigenous peoples; economic development policies; energy, education, health and rural policies; environmental protection; food security and nutrition policies; labour and employment policies; trade policies; disaster risk management (DRM) and climate change adaptation (CCA) policies fisheries access arrangements; and other fisheries sector plans, actions and investments in order to promote holistic development in small-scale fishing communities. | Responsibility of the national or local government and is therefore considered to be outside the scope of seafood certification programs. | | | |
|  |  | Special attention to ensuring gender equity and equality. | **FHR-DAP 1.1:** There is no discrimination against registered fishers or potential new program participants, particularly on the basis of race, color, sex, gender, sexual orientation, disability, marital status, family obligations, age, religion, political opinion, language, property, nationality, ethnicity or social origin, or any other condition that could give rise to discrimination in relation to: participation, rules for program participation, voting rights, the right to be elected, access to markets, access to training, technical support, or any other benefits the program offers.  **FHR-DAP 1.2:** There is no discrimination against workers, particularly on the basis of race, color, sex, gender, sexual orientation, disability, marital status, family obligations, age, religion, political opinion, language, property, nationality, ethnicity or social origin, membership of unions or other workers’ representative bodies, or any other condition that could give rise to discrimination in: recruitment, promotion, access to training, remuneration, allocation of work, termination of employment, retirement, or other activities  **ECD-DM 1:** There is a written assessment that outlines the needs of the fishers, workers, community, and the environment.  * Attention to discrimination but not specific to gender equity and equality. |  | | |
| 10.2 | | States should, as appropriate, develop and use spatial planning approaches, including inland and marine spatial planning, which take due account of small-scale fisheries interest and the role of integrated coastal zone management. Through consultation, participation, and publicizing, gender sensitive policies and laws on regulated spatial planning should be developed as appropriate. Where appropriate, formal planning systems should consider methods of planning and territorial development methods used by small-scale fishing and other communities with customary tenure systems, and decision-making processes within those communities. | Considered to be outside the scope of fishery certification programs and instead the responsibility of government authorities. | | | |
| 10.3 | | States should adopt specific policy measures to ensure the harmonization of policies affecting the health of marine and inland waterbodies and ecosystems and to ensure that fisheries, agriculture and other natural-resource policies collectively enhance the interrelated livelihoods derived from these sectors. | Considered to be outside the scope of fishery certification programs and instead the responsibility of government authorities. | | | |
| 10.4 | | States should ensure that fishery policy provides a long-term vision for sustainable small-scale fisheries and the eradication of hunger and poverty, using an ecosystem approach. | **ECD-DM 2.1:**  The certificate holder and the Fair Trade Committee together develop a written Fair Trade Premium Plan based upon the needs assessment, with the objective to meet the needs of the registered fishers, workers, community, and/or environment. It includes both short-term and long-term goals for the use of the Premium.  **RM-FD 2.3:**  The Fishery Management Plan includes a data collection strategy that: a) Incorporates both short-term and long-term data collection goals  **EDC-DM 1.2:** The needs assessment analyzes how Fair Trade may help to address those needs and any potential obstacles.  Interpretation Guidance: Where food security is a concern, the needs assessment should include an assessment of how Fair Trade can promote food security.  **WWS-CE 2**: Salaries and wages are decent, and increasing towards a living wage. | **Vision:** Our vision is of the world’s oceans teeming with life, and seafood supplies safeguarded for this and future generations.  **SA3.3.2:** Teams shall interpret the SG100 level relating to “information adequate to support a strategy” to include information provided by a strategic research plan, that addresses the information needs of management. This information shall go beyond the immediate short-term management needs to create a strategic body of research relevant to the long-term fishery-specific management system.  **Principle Indicator 3.1.3:** The management policy has clear long-term objectives to guide decision- making that are consistent with MSC Fisheries Standards, and incorporates the precautionary approach.  **Principle Indicator 3.2.1:**  The fishery- specific management system has clear, specific objectives designed to achieve the outcomes expressed by MSC’s Principles 1 and 2.  - Does not provide a framework for promoting the eradication of hunger and poverty | | |
|  |  | The overall policy framework for fisheries should be coherent with the long-term vision and policy framework for small-scale fisheries and human rights, paying particular attention to vulnerable and marginalized people. | **Fundamental Human Rights (FHR)**  **RM-FD 2.3:** The Fishery Management Plan includes a data collection strategy that:  a) Incorporates both short-term and long-term data collection goals. | **Principle Indicator 3.1.3:** The management policy has clear long-term objectives to guide decision- making that are consistent with MSC Fisheries Standard, and incorporates the precautionary approach.  **Principle Indicator 3.2.1:**  The fishery- specific management system has clear, specific objectives designed to achieve the outcomes expressed by MSC’s Principles 1 and 2.  - No particular attention to human rights and attention to vulnerable and marginalized people | | |
| 10.5 | | States should establish and promote the institutional structures and linkages- including local- national- regional- global linkages and networks- necessary for achieving policy coherence, cross-sectoral collaboration and the implementation of holistic and inclusive ecosystem approaches in the fisheries sector. At the same time, there is a need for clear responsibilities and there should be well defined points of contact in government authorities and agencies for small-scale fishing communities. | Considered to be outside the scope of scope of seafood certification programs as this requires changes within governmental structures and agencies. | | | |
| 10.6 | | Small-scale fisheries stakeholders should promote collaboration among their professional associations, including fisheries cooperatives and CSOs. | **FA: Fisher Associations**  **FTC: Fair Trade Committee**  **STR-FTC 3.1:** Each Fair Trade Committee holds a General Assembly meeting at least once a year. |  | | |
|  |  | They should establish networks and platforms for the exchange of experiences and information and to facilitate their involvement in policy and decision-making processes relevant to small-scale fishing communities. | **STR-CH 4.2:** There are regular meetings between the certificate holder and the Fisher Association(s) concerning the Fair Trade program and management of the fishery. Issues and concerns of the registered fishers and applicable workers are solicited and discussed. These meetings are documented.  **STR-CH 4.5:** At least one representative of the Fisher Association is present during meetings about corrective actions required by the Certification Body that relate to the management of the fishery.  **RM-GOV 2.2:** There is a written co-management commitment signed by the Fair Trade Committee, the certificate holder, and the agency legally responsible for the resource articulating:  a) A shared commitment to undertake resource management efforts necessary for Fair Trade certification.  b) A commitment to undertake collaborative management of the fishery. The fisheries management authorities will i) inform stakeholders of changes in political directives, ii) consult stakeholders prior to making decisions regarding management changes, and iii) commit  to providing fair feedback to the core issues raised by stakeholders.  c) A commitment by all parties to attend at least one yearly co-management meeting aimed at collaborative discussion of management improvements, particularly those with the potential to be implemented at the scale of the fishery. | **Principle Indicator 3.1.2 :**  The management system has effective consultation processes that are open to interested and affected parties.  The roles and responsibilities of organizations and individuals who are involved in the management process are clear and understood by all relevant parties.  Scoring Issue (b): Consultation processes  Scoring Issue (c): Participation  **Principle Indicator 3.2.2:** The fishery- specific management system includes effective decision- making processes that result in measures and strategies to achieve the objectives and has an appropriate approach to actual disputes in the fishery.  Scoring Issue (b): Responsiveness of decision-making processes  Scoring Issue (d): Accountability and transparency of management system and decision making process | | |
| 10.7 | | States should recognize, and promote as appropriate, that local governance structures may contribute to an effective management of small-scale fisheries. | **STR-FA 1.1:** The registered fishers are members of a Fisher Association in order to ensure democratic fisher input into decision-making about changes in the management of the fishery. | **Principle Indicator 3.1.2:** The management system has effective consultation processes that are open to interested and affected parties.  The roles and responsibilities of organizations and individuals who are involved in the management process are clear and understood by all relevant parties.  Scoring Issue (c): Participation | | |
|  |  | Taking into account the ecosystem approach and in accordance with national law. | Introduction: Goal and Objective  1) Empowerment  2) Economic Development  3) Social Responsibility  4) Environmental Stewardship  Requires compliance with all relevant local and national laws and regulations. | Ecosystem approach is not taken into account but this program is implemented in accordance with national law | | |
| 10.8 | | States should promote enhanced international, regional and sub-regional cooperation in securing sustainable small-scale fisheries. | Considered to be outside the scope of fishery certification programs. | | | |
|  |  | States, as well as international, regional and sub-regional organizations, as appropriate, should support capacity development to enhance the understanding of small-scale fisheries and assist the subsector in matters that require sub-regional, regional or international collaboration, including appropriate and mutually agreed technology transfer. | Considered to be outside the scope of fishery certification programs. | | | |
| **11. Information, research and communication** | | | | | | |
| 11.1 | | States should establish systems for collecting fisheries data, including bioecological, social, cultural, and economic data relevant for decision-making on sustainable management of small-scale fisheries with a view of ensuring sustainability of ecosystems, including fish stocks, in a transparent manner. | **RM- DC 1:** There is a system in place to collect fishery data necessary to comply with this standard.  There is criteria related to the social aspect of the fishery but there is not a system to collect related on-going data. | **Principle Indicator 1.2.3:** Relevant information is collected to support the harvest strategy.  * There is a system for collecting data related to bioecological parameters but not one for collecting social, cultural and economic data. | | |
|  |  | Efforts should be made to produce gender-disaggregated data in official statistics, as well as data allowing for an improved understanding and visibility of the importance of small-scale fisheries and its different components including socioeconomic aspects. |  |  | | |
| 11.2 | | All stakeholders and small-scale fisheries communities should recognize the importance of communication and information, which are important for effective decision-making. | **STR-CH 4.2:** There are regular meetings between the certificate holder and the Fisher Association(s) concerning the Fair Trade program and management of the fishery. Issues and concerns of registered fishers and applicable workers are solicited and discussed. These meetings are documented.  **STR-CH 4.5:** At least one representative of the Fisher Association is present during meetings about corrective actions required by the Certification Body that relate to the management of the fishery.  **STR-FA 1.4:** All major decisions of the Fisher Association are discussed and approved by members according to a free, fair, and transparent voting procedure.  **STR-FA 2.3:** Members are informed of when meetings will take place at least two weeks in advance. This includes effective communication to illiterate members  **STR-FA 2.6:** All records, books, and documentation are accessible to members of the Fisher Association. This includes effective communication to illiterate members.  **STR-FTC 2.4:** The minutes of Fair Trade Committee meetings are shared with the registered fishers. This includes effective communication to illiterate members.  **STR-FTC 3.1:** Each Fair Trade Committee holds a General Assembly meeting at least once a year.  **ECD-FTP 4.3:** The Fair Trade Committee or Fisher Association is responsible for communicating information on sales and the Fair Trade Premium to the registered fishers. This includes effective communication to illiterate members.  **ECD-FTP 4.4:** Applicable one year after the Premium Plan has been implemented: The progress of the Fair Trade Premium Plan is documented and shared with the registered fishers in an annual progress report. This includes effective communication to illiterate members. The written report includes measurements of the success against the intended outcome of all actions, and the Fair Trade Committee/board of the Fisher Association answers the following questions: • Were the projects proposed in the Fair Trade Premium Plan carried out? If not, why? • When were the projects carried out? • At what cost? • Was the objective achieved or is further action needed? | **Principle Indicator 3.1.2:** The management system has effective consultation processes that are open to interested and affected parties.  The roles and responsibilities of organizations and individuals who are involved in the management process are clear and understood by all relevant parties.  Scoring Issue (b): Consultation processes  Scoring Issue (c): Participation  **Principle Indicator 3.2.2:**  The fishery specific management system includes effective decision-making processes that result in measures and strategies to achieve the objectives and has an appropriate approach to actual disputes in the fishery.  SCORING ISSUE (b): Responsiveness of decision-making processes  SCORING ISSUE (d): Accountability and transparency of management system and making process | | |
| 11.3 | | States should endeavor to prevent corruption, particularly through increasing transparency, holding decision-makers accountable and ensuring that impartial decisions are delivered promptly and through appropriate communication with small-scale fishing communities. | **STR-CH 4.2:** There are regular meetings between the certificate holder and the Fisher Association(s) concerning the Fair Trade program and management of the fishery. Issues and concerns of the registered fishers and applicable workers are solicited and discussed. These meetings are documented.  **STR-CH 4.4:** Cost breakdowns of inputs and services are available, transparent, and coherent. Charges for inputs and services are agreed upon in advance. Costs of inputs and services are not higher than normal market prices.  **STR- CH 4.5:**  At least one representative of the Fisher Association is present during meetings about corrective actions required by the Certification Body that relate to the management of the fishery.  **STR- FA 1.1:** The registered fishers are members of a Fisher Association in order to ensure democratic fisher  input into decision-making about changes in the management of the fishery.  **STR- FA 1.4:**  All major decisions of the Fisher Association are discussed and approved by members according to a free, fair, and transparent voting procedure.  **STR-FA 2:**  Fisher Association meetings adhere to agreed-upon rules, and communication and management of those meetings is effective.  **STR- FTC 1.2:**  The Fair Trade Committee is chosen in free, fair, and transparent elections and this election process is documented.  **STR-FTC 1.7:** The internal rules of the Fair Trade Committee (i.e., statutes, constitution, or by-laws) mandate all members have decision-making voting rights.  **STR-FTC 1.8:** Where delegate/representation systems are applied, these are clearly defined and offer equitable representation to all members of the organization.  **STR- FTC 3.5:** All major decisions of the Fair Trade Committee are discussed and approved at a General  Assembly meeting according to a free, fair, and transparent voting procedure.  **RM-GOV 2.2:** There is a written co-management commitment signed by the Fair Trade Committee, the certificate holder, and the agency legally responsible for the resource articulating:  a) A shared commitment to undertake resource management efforts necessary for Fair Trade certification.  b) A commitment to undertake collaborative management of the fishery. The fisheries management authorities will i) inform stakeholders of changes in political directives, ii) consult stakeholders prior to making decisions regarding management changes, and iii) commit to providing fair feedback to the core issues raised by stakeholders. c) A commitment by all parties to attend at least one yearly co-management meeting aimed at collaborative discussion of management improvements, particularly those with the potential to be implemented at the scale of the fishery. | **Principle Indicator 3.2.2:** The fishery specific management system includes effective decision-making processes that result in measures and strategies to achieve the objectives and has an appropriate approach to actual disputes in the fishery.  Scoring Issue (d): Accountability and transparency of management system and making process | | |
| 11.4 | | All parties should recognize small-scale fishing communities as holders, providers and receivers of knowledge. It is particularly important to understand the need for access to appropriate information by small-scale fishing communities and their organizations in order to help them cope with existing problems and empower them to improve their livelihoods. These information requirements depend on current issues facing communities and concern the biological, legal, economic, social and cultural aspects of fisheries and livelihoods. | Holders and Providers  **RM-FD 2.4:** The following are discussed during a General Assembly meeting and included in the Fishery Management Plan: a) Incentives: Historical and current incentives that contribute to problematic fishing patterns to better understand the fishery. b) Behavioural solutions: Fishers suggest acceptable methods for improving fishery management. c) Innovations: Fishers propose innovations for improving fishery management. d) Examples: Alternative solutions from similar fisheries are researched if no suggestions are made on b or c. e) Action Plan: An action plan with timelines, activities and personnel assigned to each activity is created. Assignees agree to activities assigned. Numeric targets are identified.  **RM-FD 2.7:** A map depicting the fishing range of registered fishers has been developed using available information from local or national agencies and local fishers’ knowledge. The map includes:  a) The fishing range of registered fishers  b) The spatial distribution of different habitat types, both inside and outside fishing areas  c) Benthic information, such as bathymetry  Receivers  **STR-CH 4.6:** The Certificate holder and Fisher Association take measures to improve the registered fishers’ understanding of financial management and to increase their knowledge on pricing and international market mechanisms.  **WWS-OH 3:** Individuals have the training and information they need to keep themselves safe.  **RM-DC 2.3:** Data collectors, including fishers, are regularly trained in data collection, data safety, and data management. Written procedures are provided regarding these topics.  **STR-CH 4.1:** Corporate social responsibility and the empowerment of registered fishers is an integral part of the certificate holder’s written mission or policy statement(s). | Holders and Providers  **Principle Indicator 3.1.2:** The management system has effective consultation processes that are open to interested and affected parties.  The roles and responsibilities of organizations and individuals who are involved in the management process are clear and understood by all relevant parties.  Scoring Issue (b) Consultation  **Principle Indicator** 3.2.2 The fishery specific management system includes effective decision-making processes that result in measures and strategies to achieve the objectives and has an appropriate approach to actual disputes in the fishery.  Scoring Issue (b) (b) Responsiveness of decision-making processes | | |
| 11.5 | | States should ensure that the information necessary for responsible small-scale fisheries and sustainable development is available including on illegal, unreported and unregulated (IUU) fishing. | **RM-GOV 1:** Illegal fishing is monitored and reported. | **Box GSA2: IUU fishing** | | |
|  |  | It should relate to inter alia, disaster risks, climate change, livelihoods and food security with particular attention to the situation of vulnerable and marginalized groups. | **ECD-DM 1**: There is a written assessment that outlines the needs of the fishers, workers, community and the environment.  EDC - DM 1.2: The needs assessment analyzes how Fair Trade may help to address those needs and any potential obstacles.  Guidance: Where food security is a concern, the needs assessment should include an assessment of how Fair Trade can promote food security.  - Does not directly consider climate change, disaster risks however, these issues could be identified in the needs assessment for the use of the Fair Trade Premium. |  | | |
|  |  | Information systems with low data requirements should be developed for data-poor situations. | **Appendix B: Data-Limited Stock Assessment Decision Tree**  - Related to responsible small-scale fisheries but not to the other specific categories outlined in 11.5 | **Risk-Based Management Framework**  - Related to responsible small-scale fisheries but not to the other specific categories outlined in 11.5 | | |
| 11.6 | | All parties should ensure that the knowledge, culture, traditions and practices of small-scale fishing communities, including Indigenous peoples, are recognized and, that they inform responsible local governance and sustainable development processes. | **RM-FD 2.4:** The following are discussed during a General Assembly meeting and included in the Fishery Management Plan: a) Incentives: Historical and current incentives that contribute to problematic fishing patterns to better understand the fishery. b) Behavioural solutions: Fishers suggest acceptable methods for improving fishery management. c) Innovations: Fishers propose innovations for improving fishery management. d) Examples: Alternative solutions from similar fisheries are researched if no suggestions are made on b or c. e) Action Plan: An action plan with timelines, activities and personnel assigned to each activity is created. Assignees agree to activities assigned. Numeric targets are identified.  **RM-FD 2.7:** A map depicting the fishing range of registered fishers has been developed using available information from local or national agencies and local fishers’ knowledge. The map includes:  a) The fishing range of registered fishers  b) The spatial distribution of different habitat types, both inside and outside fishing areas  c) Benthic information, such as bathymetry  * Knowledge of local fishers informs responsible local governance and sustainable development processes but culture, traditions and practices are not mentioned    * It is important to note that small-scale fishing communities and Indigenous peoples cannot be equated registered fishers in all cases. | **Principle Indictor** **3.1.1** The management system exists within an appropriate and effective legal and/or customary framework which ensures that it:  - Is capable of delivering sustainability in the UoA(s)  - Observes the legal rights created explicitly or established by custom of people dependent on fishing for food or livelihood; and  -Incorporates an appropriate dispute resolution framework.  **Principle Indicator 3.1.2:** The management system has effective consultation processes that are open to interested and affected parties. The roles and responsibilities of organizations and individuals who are involved in the management process are clear and understood by all relevant parties.  Scoring Issue (b) Consultation  * It is important to note that small-scale fishing communities and Indigenous peoples cannot be equated certified fishers in all cases. | | |
|  |  | The specific knowledge of women fishers and fish workers must be recognized and supported. | **FHR-DAP 1.1:** There is no discrimination against registered fishers or potential new program participants, particularly on the basis of race, color, sex, gender, sexual orientation, disability, marital status, family obligations, age, religion, political opinion, language, property, nationality, ethnicity or social origin, or any other condition that could give rise to discrimination in relation to: participation, rules for program participation, voting rights, the right to be elected, access to markets, access to training, technical support, or any other benefits the program offers.  - There cannot be discrimination against the sex of a program participant however, no specific mention of knowledge |  | | |
|  |  | States should investigate and document traditional fisheries knowledge and technologies in order to access their application to sustainable fisheries conservation, management and development. |  |  | | |
| 11.7 | | States and other relevant parties should provide support to small-scale fishing communities, in particular to Indigenous peoples, women and those that rely on fishing for subsistence, including, as appropriate, the technical and financial assistance to organize, maintain, exchange and improve traditional knowledge of aquatic living resources and fishing techniques and upgrade knowledge on aquatic ecosystems. | **Fisher Association**  **RM-GV 2:**  The Fisher Association is actively involved in the management of the fishery.  **RM-DC 2.3:** Data collectors, including fishers, are regularly trained in data collection, data safety, and data management. Written procedures are provided regarding these topics.  **RM-FD 2.4:** The following are discussed during a General Assembly meeting and included in the Fishery Management Plan:  a) Incentives: Historical and current incentives that contribute to problematic fishing patterns to better understand the fishery.  b) Behavioural solutions: Fishers suggest acceptable methods for improving fishery management.  c) Innovations: Fishers propose innovations for improving fishery management. d) Examples: Alternative solutions from similar fisheries are researched if no suggestions are made on b or c.  e) Action Plan: An action plan with timelines, activities and personnel assigned to each activity is created. Assignees agree to activities assigned. Numeric targets are identified.  * Not specifically for Indigenous peoples, women or those that rely on fishing for subsistence | |  | |
| 11.8 | | All parties should promote the availability, flow and exchange of information, including on aquatic transboundary resources, through the establishment or use of appropriate existing platforms and networks at community, national, sub-regional, and regional level, including both horizontal and vertical two-way information flows. | Considered out of the scope of fishery certification programs. | | | |
|  |  | Taking into account the social and cultural dimensions, appropriate approaches, tools and media should be used for communication with and capacity development for small-scale fishing communities. | Capacity development is part of the FT USA standard and social dimensions are included. |  | | |
| 11.9 | | States and other parties involved should, to the extent possible, ensure that funds are available for small-scale fisheries research, and collaborative and participatory data collection, analyses and research should be encouraged. | **RM-DC 1.1:** A data collection system is in place and accessible to all registered fishes and resource managers. This includes effective communication to illiterate members.  **RM-DC 1.3:** Vessels record fishing trip data with crew lists for each voyage. Records shall include:  a) Date of trip  b) Fishing location  c) Time out/time in  d) Port/Landing site  e) Vessel name  f) Captain(s)/skipper name(s)  g) Crew names, ages, and titles if relevant  h) Fishing licenses held by fishers in crew  i) Whether fishing by share-catch agreement  j) Type of gear used  **RM-DC 1.4:** Fishing trips record catch data on primary and secondary species by:  a) Species  b) Cumulative landed weight for each species  c) The total length of individuals of each species  d) ETP classification of each species, where appropriate  Fishing trips record catch data on bycatch species by:  a) Species  b) Number of individuals of each species  c) ETP classification for each species, where appropriate  **RM-DC 1.6:** Catch data are reviewed every six months for accuracy. Where necessary, adjustments are made to the data collection strategy (both collection of data and data entry) to ensure the data are accurate.  Interpretation Guidance: Such adjustments may include additional educational training for fishers on data collection and documentation.  **RM-DC 2:** Data collectors, including fishers, are regularly trained in data collection, data safety (including backups), and data management. Written procedures are provided regarding the above topics.  - Research and analyses are not explicitly outlined in the standard | **MSC Global Fisheries Sustainability Fund**  - Does not explicitly state that participatory data collection and analyses are encouraged | | |
|  |  | States and other parties should endeavor to integrate this research knowledge into decision-making processes. | The data collected above informs the fisheries management plan and compliance standard requirements. |  | | |
|  |  | Research organizations and institutions should support capacity development to allow small-scale fisheries communities to participate in research and in the utilization of research findings. |  | The **Global Fisheries Sustainability Fund** is aimed at strengthening knowledge and global capacity to assist small-scale and developing world fisheries in their journey to achieving MSC certification (MSC, 2015).  - Utilization of the research in the sense that it will increase ability to become MSC certified. Not explicit mention of their participation in this research. | | |
|  |  | Research priorities should be agreed upon through a consultative process focusing on the role of small-scale fisheries in sustainable resource utilization, food security and nutrition, poverty eradication, and equitable development, including also DRM and CCA considerations. |  |  | | |
| 11.10 | | States and other relevant parties should promote research into the conditions of work, including of migrant fishers and fish workers, and inter alia health, education, decision-making, in the context of gender relations in order to inform strategies of ensuring equitable benefits for men and women in fisheries. Efforts to mainstream gender should include the use of gender analysis should be used in the design of policies, programmes and projects for small-scale fisheries in order to design gender sensitive interventions. Gender-sensitive indicators should be used to monitor and address gender inequalities and to capture how interventions have contributed towards social change. |  |  | | |
| 11.11 | | Recognizing the role of small-scale fisheries in seafood production. | Standard developed to bring the benefits of Fair Trade to fishers and their communities. Several of the certified fisheries are considered small-scale. | Small-scale fisheries can participate in the MSC certification program. | | |
|  |  | States and other parties should promote the consumption of fish and fishery products within consumer education programmes in order to increase awareness of the nutritional benefits of eating fish and impart knowledge on how to assess the fish and fishery product quality. | Seafood certification programs, including Fair Trade, act as consumer education programs by identifying a product deemed “sustainable” against a specific standard. This certification does not directly increase awareness of the nutritional benefits of eating fish and how to assess the fish and fishery product quality. However, the ecolabel could be indication of product quality. | Seafood certification programs, including MSC, act as consumer education programs by identifying a product deemed “sustainable” against a specific standard. This certification does not directly increase awareness of the nutritional benefits of eating fish and how to assess the fish and fishery product quality. However, the ecolabel could be indication of product quality. | | |
| **12. Capacity development** | | | | | | |
| 12.1 | | State and other parties should enhance the capacity of small-scale fishing communities in order to enable them to participate in decision-making processes. | The Fishery Association  **STR-FA 1.1:** The registered fishers are members of a Fisher Association in order to ensure democratic fisher input into decision-making about changes in the management of the fishery.  **STR-FA 1.3:** The internal rules of the Fisher Association (i.e., statutes, constitution, or by-laws) mandate that all members have decision-making voting rights.  **STR-FA 1.4:** All major decisions of the Fisher Association are discussed and approved by members according to a free, fair, and transparent voting procedure.  The Fair Trade Committee  **STR-FTC 3.5:** All major decisions of the Fair Trade Committee are discussed and approved at a General Assembly meeting according to free, fair and transparent voting procedure.  **RM- GOV 2:** The Fisher Association is actively involved in the management of the fishery. |  | | |
|  |  | It should be ensured that the range and diversity of the small-scale fisheries subsector along the entire value chain is appropriately represented through the creation of legitimate, democratic and representative structures. | **RM- GOV 2:** The Fisher Association is actively involved in the management of the fishery.  **RM-GOV 2.2:** There is a written co-management commitment signed by the Fair Trade Committee, the certificate holder, and the agency legally responsible for the resource.  * This does not include the entire value chain. |  | | |
|  |  | Specific attention should be paid towards the equitable participation of women in such structures. Where appropriate and necessary, separate spaces and mechanisms should be provided to enable women to organize autonomously at various levels on issues of particular relevance to them. |  |  | | |
| 12.2 | | States and other stakeholders should provide capacity building, for example through development programsmes, to allow small-scales fisheries to benefit from market opportunities. | **STR-CH 4.6:** The certificate holder and Fisher Association take measures to improve the registered fishers’ understanding of financial management and to increase their knowledge on pricing and international market mechanisms.  **ECD-FTP 4:** Communication about Fair Trade sales and use of the Fair Trade Premium is thorough and consistent.  **ECD-FTP 5.4:** The certificate holder provides trainings to the Fair Trade Committee covering administrative  and organizational capacity-building and management of the Fair Trade Premium, including the accounting system. All trainings are documented. Records are kept on file. | MSC Capacity Building Program which includes:  - Fishery improvement tools  - Capacity building toolkit  - Capacity building training workshop  - Risk-based framework | | |
| 12.3 | | All parties should recognize that capacity development should build on existing knowledge and skills. | **RM-DC 1.6:** Catch data are reviewed every six months for accuracy. Where necessary, adjustments are made to the data collection strategy (both collection of data and data entry) to ensure the data are accurate.  Interpretation Guidance: Information is assumed to be either available through local knowledge, or through existing gray, white or primary literature publications. | The MSC standard builds off of existing knowledge of the fishery. There is not focus on building on existing skills. | | |
|  |  | Two-way process of knowledge transfer, providing for flexible and sustainable learning pathways to meet the needs of individuals, including both men and women and vulnerable and marginalized groups. | **RM- GOV 2:** The Fisher Association is actively involved in the management of the fishery.  **FHR-DAP 1:** There is no discrimination against registered fishers, potential new program participants, or workers. |  | | |
|  |  | Capacity development should include building the resilience and adaptive capacity of small-scale fishing communities in relation to DRM and CCA. | Considered outside the scope of seafood certification programs. | | | |
| 12.4 | | Government authorities and agencies at all levels should work to develop knowledge and skills to support sustainable small-scale fisheries development and successful co-management arrangements, as appropriate. | **RM-GOV 2:** The Fisher Association is actively involved in the management of the fishery. |  | | |
|  |  | Particular attention should be given to decentralized and local government structures directly involved in governance and development processes together with SSF communities, including the area of research. | **Fisher Association**  **Fair Trade Committee**  **RM-GOV 2:** The Fisher Association is actively involved in the management of the fishery.  * Research is not a component of the Fair Trade standard |  | | |
| **13. Implementation Support and Monitoring** | | | | | | |
|  | | | | | | |
